# Promotion of influenza vaccination among health care workers: findings from a tertiary care children’s hospital in Italy

**DOI:** 10.1186/s12889-015-2067-9

**Published:** 2015-07-24

**Authors:** Vanessa Cozza, Valeria Alfonsi, Maria Cristina Rota, Valerio Paolini, Marta Luisa Ciofi degli Atti

**Affiliations:** Department of Medical and Surgical Sciences, University of Foggia, Foggia, Italy; Medical Direction, Bambino Gesù Children’s Hospital, Rome, Italy; Istituto Superiore di Sanità (ISS), National Centre for Epidemiology Surveillance and Health Promotion, Rome, Italy; Clinical Epidemiology Unit, Medical Direction, Bambino Gesù Children’s Hospital, Piazza Sant’Onofrio, 4, Rome, 00165 Italy

**Keywords:** Influenza vaccine, Healthcare workers, Communication campaign, Attitudes

## Abstract

**Background:**

The aims of this study were: a) to evaluate attitudes and practices of health care workers (HCWs) towards influenza vaccination and their opinion regarding a vaccination promotion toolkit; b) to estimate hospital HCWs’ influenza vaccination coverage rates (VC).

**Methods:**

The Bambino Gesù Children’s Hospital (OPBG) is an academic hospital in Italy. Since 2009, free influenza vaccination is offered to HCWs during working hours. In October-December 2013, a communication campaign based on a standardized toolkit was conducted. In December 2013, we performed a cross-sectional survey in a sample of hospital wards, based on a self-administered questionnaire including participants’ characteristics; self-reported influenza vaccination history; reasons for vaccination or missed vaccination; opinion regarding the toolkit. Multivariable logistic analysis was used to assess independent predictors of influenza vaccination status. Annual VC for years 2009–2013 was estimated by using the number of seasonal influenza vaccine doses administered to HCWs as numerator, and the number of hospital HCWs as denominator.

**Results:**

Out of 191 HCWs who participated in the survey, 35.6 % reported at least one influenza vaccination during their life; 6.8 % adhered to annual revaccination. Years of service and professional category were significantly and independently associated with vaccination (adjusted-OR: 2.4 for > 10 years of service, compared to < 5 years of service; adjusted-OR: 2.6 for physicians compared to nurses). Patient protection was the main reported reason for vaccination (34.3 %); considering influenza a mild disease was the main reason for non-vaccination (36.9 %); poor vaccine effectiveness was the main reason for missed annual revaccination (28.8 %). Overall, 75 % of respondents saw at least one promotion tool; 65.6 % of them found the information useful. Hospital VC decreased from 30 % in 2009, to 5 % in 2012. In 2013, VC was 14 %.

**Conclusions:**

Satisfactory influenza VC in HCWs is hard to achieve. In 2013, along with the toolkit implementation, we observed an increase in HCWs’ vaccination coverage, nevertheless, it remained unsatisfactory. Tailored information strategies targeting nurses and recently employed HCWs should be implemented. Institution of declination statements, adding influenza vaccination to financial incentive systems, or vaccination requirements should also be considered to increase influenza VC among HCWs.

## Background

Vaccination against seasonal influenza is recommended to health care workers (HCWs) by national and international institutions [1–4], in order to reduce the risk of acquiring influenza and transmitting the infection to vulnerable patients [5–8]. Influenza outbreaks may also cause HCWs’ absenteeism, resulting in inadequate staffing, diminished quality of care and increased costs [9, 10].

A recent meta-analysis confirmed that HCWs’ influenza vaccination is effective in preventing mortality and influenza cases among patients of healthcare facilities [11]. However, influenza vaccination coverage among HCWs remains suboptimal worldwide [12–15]. In Europe, data from 10 countries for the 2010–2011 influenza season showed a vaccination coverage <35 % in 8 countries, and ranging from 41 to 64 % in the remaining two countries [14].

In Italy, influenza vaccination of HCWs is recommended by the Ministry of Health and is offered free of charge by the national health service (NHS) [4]. However, data on vaccination coverage (VC) among HCWs are not routinely collected and the few ad-hoc studies have consistently found low coverage rates (12-34 %), also during the 2009–2010 pandemic [16–18]. Misperceptions about the severity of influenza, lack of knowledge on the benefits of the vaccination and fear of adverse events are frequently reported as reasons for missed vaccination [12, 16, 18]. Perceived lack of leadership support is also a potential barrier to HCWs’ influenza vaccination [12].

In 2011, the European Commission funded the HProImmune project [19], aiming at increasing awareness among HCWs of several vaccine preventable diseases, enhancing their knowledge on immunization and promoting vaccinations. The HProImmune consortium comprised 10 associated partners from 7 countries (Greece, Romania, Poland, Lithuania, Italy, Cyprus and Germany) and 2 European collaborating partners (WHO/EURO Centre for Environment and Health and Health Protection Agency - UK).

Within this project, influenza vaccination was identified as a priority and a toolkit for immunization promotion was developed on the basis of healthcare personnel’s needs and perspectives, as identified through a literature review and through qualitative methods (i.e. focus groups) exploring behaviours and barriers towards immunization. In Autumn 2013, the Bambino Gesù Children’s Hospital, the largest Italian children’s hospital, used the HProImmune influenza toolkit to promote influenza vaccination for HCWs.

In this article, we present: a) the results of a survey conducted among HCWs to assess attitudes and practices regarding influenza vaccination, and opinion regarding the toolkit; b) estimates of the hospital’s HCWs’ vaccination coverage rates for influenza from 2009 to 2013.

## Methods

### Vaccination offer and communication campaign

The Bambino Gesù Children’s Hospital (OPBG) is a tertiary care academic hospital, with 607 inpatient-bed, located in Rome, Italy. Since 2009, free influenza vaccination is offered to HCWs through mobile teams and dedicated vaccination sessions, from October to December. Information about the vaccination offer is sent to all hospital wards and posted on the hospital’s intranet. The number of influenza vaccine doses administered to HCWs is recorded by the Medical direction. In October-December 2013, a communication campaign based on the HproImmune influenza toolkit was conducted. The communication tools consisted in: i) posters, placed in areas which were frequently visited by HCWs (changing rooms, attendance recorder area, canteen, etc.); ii) information factsheets, distributed in paper form in hospital wards and also available in electronic format on the hospital’s intranet; iii) a banner on the hospital’s intranet, linked to the electronic information factsheets. The campaign’s key messages focused on personal protection and patient protection (i.e., “Protect your patients! Protect yourself!”, “Be prepared. Get vaccinated!”, “Get informed! Get vaccinated! Get protected!”).

### Cross sectional survey

In December 2013, we assessed the HCWs’ attitudes and practices towards seasonal influenza vaccination and their opinion regarding the communication campaign. The study population included physicians, nurses, other HCWs (including students and trainees). We performed a cross-sectional survey in all intensive care units (*n* = 5) and in a random sample of 21 % of the hospital non-critical inpatient medical and surgical units (*n* = 7/33). Overall, a total of 12 wards were included in the study.

During the day shift of selected days, we invited HCWs to fill in an anonymous, self-administered paper questionnaire, including the following information: age group (<35, 35–44, 45–54, ≥ 55 years); professional category (physician, nurse, other profession); type of ward (intensive care, medical, surgical); years of service (<5, 5–10, >10); self-reported influenza immunization history (having ever received the vaccination); year of first influenza vaccination (<2008; 2008–2011; >2011); adherence to annual revaccination; main reason for vaccination, missed vaccination, and missed annual revaccination; opinion regarding the communication campaign (having seen the tools, usefulness, perceived key messages).

### Statistical analysis

Data were entered in a dedicated database. We performed a descriptive analysis of socio-demographic and professional characteristics, vaccination history, attitude towards and uptake of influenza vaccine, and exposure to the toolkit. Proportions were calculated excluding missing values. The Chi-squared or Fisher exact tests were used for univariate analyses with *p* < 0.05 being considered statistically significant. The Odds Ratios (ORs) with 95 % confidence intervals (95 %CI) were used to evaluate the association between socio-demographic and professional characteristics and having ever been vaccinated against influenza. Covariates identified as potential predictors of influenza vaccination at the univariate analysis were considered as candidates for the multivariable analysis. Logistic regression was used to assess independent predictors of self-reported influenza vaccination; the outcome of interest was having ever been vaccinated against influenza or not. The analysis was performed using the STATA software v. 12.

### Seasonal influenza vaccination rate

We evaluated the annual seasonal influenza vaccination coverage rates (VC) at the hospital level, from 2009 to 2013. Annual coverage rates were estimated by using the number of seasonal influenza vaccine doses administered to HCWs as the numerator, and the number of OPBG HCWs as the denominator. Being the OPBG’s HCWs’ population stable over the years, we used the 2013 data as reference (1813 HCWs including 619 physicians, 1075 nurses and 119 other HCWs).

The study was approved by the Hospital Committee for Infection Control; there was no intervention on participants, and written informed consent was not required.

## Results

A total of 191 HCWs participated in the survey; the response rate was 90.8 % (109/120) for nurses and 83.7 % (41/49) for physicians. Other HCWs who were on duty in the selected units in the days of the survey responded to the questionnaire (i.e. personnel dedicated to more than one unit or volunteering); due to the uncertainty of the denominator, it was not possible to calculate the response for these professional categories. Most respondents were nurses (57 %), worked in intensive care units (53.4 %), and had more than 10 years of service (43.7 %) (Table 1). Overall, 68 (35.6 %) respondents reported at least one influenza vaccination during their life; the majority of them received their first influenza vaccine between 2008 and 2011 (49/68; 72.1 %). Only 6.8 % of all participants (13/191), and 19.1 % of participants previously vaccinated (13/68) reported to adhere to annual revaccination.

**Table 1:** Characteristics of HCWs, self-reported vaccination history and opinion regarding the communication campaign; OPBG, 2013 (Number of participants: 191; number of respondents to each item are reported in brackets)

	Number	%
Age group, years *(n = 190)*		
< 35	74	39.0
35-44	58	30.5
45-54	46	24.2
≥ 55	12	6.3
Professional category *(n = 191)*		
Nurse	109	57.0
Physician	41	21.5
Other HCWs	41	21.5
Type of ward *(n = 191)*		
Intensive care	102	53.4
Medical	56	29.3
Surgical	33	17.3
Years of service *(n = 190)*		
< 5	50	26.3
5 – 10	57	30.0
> 10	83	43.7
Ever vaccinated against influenza	68	35.6
*If yes, year of first influenza vaccination (n = 68)*		
*< 2008*	15	*22.1*
*2008-2011*	49	*72.1*
*> 2011*	4	*5.8*
*If yes, adherence to annual revaccination (n = 68)*	13	*19.1*
Exposure to at least one communication tool	144	75.4
Usefulness of communication campaign *(n = 142)*		
Yes	93	65.5
No	20	14.1
Do not know	29	20.4
Key perceived message *(n = 139)*		
Risk of influenza transmission to patients	64	46.0
Vaccination is important/Influenza is dangerous	42	30.2
Others	4	2.9
Do not know	29	20.9

Approximately 75 % (144/191) of participants had seen at least one promotion tool; 65.5 % of the respondents who had seen the tools (93/142) found the information useful. The main perceived message for promoting vaccination was risk of influenza transmission from HCWs to patients (46 % of respondents) (Table 1). Perceived key messages did significantly vary by vaccination status; vaccinated HCWs reported importance of vaccination and influenza risks as the main key message 2.6 times more frequently than unvaccinated colleagues (25/68, vs 17/122; *p* < 0.001) (data not shown in the table).

Patient protection was the main reason for vaccination (34.3 %); considering influenza as a mild disease was the main reason for non-vaccination (36.9 %); poor vaccine effectiveness was the main reported reason for missed annual revaccination (28.8 %) (Table 2).

**Table 2:** Reported reasons for vaccination, missed vaccination and missed annual revaccination among HCWs; OPBG, 2013 (Number of respondents to each item are reported in brackets)

**Reasons for vaccination ** ***(n = 67)***	**Number among vaccinated (%)**
Vaccination is important to protect patients	23 (34.3)
The risk to contract flu in the hospital is high	18 (26.9)
To avoid illness and sick-leave	15 (22.4)
Vaccine is offered free of charge at OPBG	4 (6.0)
I am affected with a chronic condition	3 (4.4)
Influenza is a disease potentially severe	2 (3.0)
Other reasons	2 (3.0)
**Reasons** **for ** **missed vaccination ** ***(n = 122)***	**Number among not vaccinated (%)**
Influenza is a mild disease	45 (36.9)
I do not believe in vaccination practice	24 (19.7)
I am afraid of adverse events	19 (15.6)
I am not informed enough on the benefits	14 (11.5)
Vaccination was not proposed to me	9 (7.4)
I forgot / I did not have time	4 (3.2)
Influenza vaccine is not effective	3 (2.5)
Other reasons	4 (3.2)
**Reasons ** **for missed annual revaccination ** ***(n = 52)***	**Number among not revaccinated (%)**
Vaccine was not effective	15 (28.8)
Adverse events appeared	11 (21.2)
Forgetfulness or lack of time	10 (19.2)
Change of attitude after the pandemic H1N1 experience	9 (17.3)
Vaccination was not proposed to me	3 (5.8)
Other reasons	4 (7.7)

At the univariate analysis, the proportion of HCWs who reported to be vaccinated against influenza significantly varied by age, professional category, and years of service (Table 3). Results of the multivariate analysis show that years of service and professional category were significantly and independently associated with vaccination (OR: 2.4 for > 10 years of service, compared to < 5 years of service; OR: 2.6 for physicians compared to nurses).

**Table 3:** Predictors of self-reported influenza vaccine history among HCWs; OPBG, 2013

	Ever vaccinated		Univariate analysis		Multivariable logistic model	
	Yes	No	Unadjusted OR (95 % CI)	*p*-value	Adjusted OR (95 % CI)	*p*-value
**Age group (years)**					NS	
<35	20 (27.0)	54 (73.0)	Ref			
35 – 44	24 (41.4)	34 (58.6)	1.9 (0.9 – 4.0)	0.837		
45 – 54	16 (34.8)	30 (65.2)	1.4 (0.6 – 3.2)	0.369		
≥55	8 (66.7)	4 (33.3)	5.4 (1.4 – 21.3)	0.007		
**Professional category**						
Nurse	33 (30.3)	76 (69.7)	Ref		Ref	
Physician	21 (51.2)	20 (48.8)	2.4 (1.1 – 5.1)	0.018	2.6 (1.2 – 5.7)	0.014
Other profession	14 (34.2)	27 (65.8)	1.2 (0.6 – 2.6)	0.650	1.2 (0.5 – 2.6)	0.664
**Type of ward**					NS	
Intensive care	31 (30.4)	71 (69.6)	Ref			
Medical	22 (39.3)	34 (60.7)	1.5 (0.7 – 2.9)	0.259		
Surgical	15 (45.5)	18 (54.5)	1.9 (0.8 – 4.3)	0.114		
**Years of service**						
<5	12 (24.0)	38 (76.0)	Ref		Ref	
5 – 10	24 (42.1)	33 (57.9)	2.3 (1.0 – 5.4)	0.05	2.2	0.071
>10	32 (38.5)	51 (61.5)	1.9 (0.9 – 4.4)	0.09	2.4	0.040

*NS* not significant

**Fig. 1:**
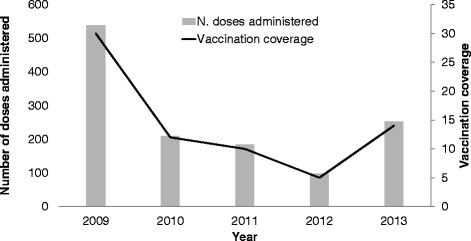
Number of influenza vaccine doses and vaccination coverage by year; OPBG, 2009–2013

At the hospital level, the number of doses of seasonal influenza vaccine administered to HCWs decreased from 539 (VC: 30 %) in 2009, to 209 (VC: 12 %) in 2010, 184 (VC: 10 %) in 2011 and 98 (VC: 5 %) in 2012. In 2013, 253 doses were administered (VC: 14 %) (Fig. 1).

## Discussion

Influenza vaccination coverage rarely exceeded 40 % in Europe between 2008 and 2011 [14], and our study confirms that satisfactory vaccination coverage rates in HCWs are hard to achieve. According to our results, only 36 % of HCWs reported to have received influenza vaccination at least once during their life. Different factors may contribute to the HCWs’ decision to receive the influenza vaccine, as reported in several studies [20–23]. In a literature review [24], self-protection was reported to be the strongest motivation of HCWs, while protecting the patient was a secondary motivation in most cases. On the contrary, in our study, 34 % of HCWs put patient protection before self-protection, while protecting themselves was reported as the main reason by 22 % of HCWs only. Determinants of HCWs’ vaccination uptake included individual factors such as knowledge, awareness of the risk of exposure to seasonal influenza in the hospital setting and responsibility towards patients regarding the risk of influenza transmission. On the other hand, misconceptions about severity of influenza, lack of knowledge on the benefits of the vaccination, lack of time, doubts about effectiveness and concern about side-effects may all play a role in refusal. Our results confirm that the professional category is a significant and independent predictor of vaccination [22]. Among different professionals, only physicians had a vaccination rate higher than 50 %. Similar findings were reported in a meta-analysis, showing that being a physician increased the chances of being vaccinated, while being a nurse was associated with a lower vaccination rate [25].

The lower adherence to influenza vaccination reported by nurses compared to physicians is a reason of concern, as nurses have closer and more frequent contacts with patients. Thus, non vaccinated nurses may increase the risk of in-hospital influenza transmission. As reported by other authors [22], another significant determinant of vaccination was years of service, with a lower adherence in HCWs with <5 working years (24 %), and in those below 35 years of age (27 %), who should be properly educated regarding the risk of influenza in the healthcare setting.

The 2009 A/H1N1 pandemic influenza experience might have played a role in decreasing the compliance to vaccination during the last seasons [26]. Few HCWs adhered to annual revaccination; among those who did not adhere, 17 % reported that their perception regarding the vaccination changed after the pandemic season. Moreover, hospital data show a sharp decrease in vaccination coverage from 2009 onward.

Misconceptions about influenza and influenza vaccine can be addressed by information and education [12]. To this regard, the HProImmune communication toolkit was positively evaluated by responding HCWs; however, our results show that the key messages regarding the importance of vaccination and influenza risks were perceived more strongly by those who were previously vaccinated.

The study population was represented by HCWs from all hospital Intensive Care Units and from a random sample of other in-patient wards. Participation rate among physicians and nurses was > 80 %, but we cannot exclude that HCWs who refused participation had different attitudes and practices towards influenza vaccination. Also, the survey did not target HCWs from outpatient clinics, who may take care of children with minor illnesses and may have different opinions regarding reasons for vaccination, such as protecting patients. Even taking into account these limitations, our results suggest that vaccine promotion should take into account specific information and communication strategies, addressing concerns of HCWs who were never vaccinated and aiming at reinforcing the value of immunization for protecting their health. Tailored educational programs for nurses should also be implemented, and influenza vaccination should be included in the initial orientation programs of all HCWs.

The benefits of HCWs’ influenza vaccination have been repeatedly shown and its promotion among HCWs is recognized as a public health priority [27–29]. In OPBG, the hospital vaccination coverage rate reached a maximum of 30 % in 2009, and was as low as 5 % in 2012, when a precautionary recall was issued in Italy for two vaccine formulations, due to the identification of visible protein aggregates in one batch [30]. Such recall caused a shortage of influenza vaccines, as well as concerns regarding vaccine safety. In 2013, vaccination coverage reported by other Authors for Italian HCWs returned to rates observed prior to 2012 [18]. In coincidence with the implementation of the toolkit, we observed an increase of adherence to vaccination, which reached the highest coverage rate since 2009, though remaining clearly suboptimal.

## Conclusions

Despite a strategy that included free vaccination offer during working hours, easy access to vaccine, and use of a standardised communication toolkit, the hospital influenza vaccination programme has been poorly effective. Interventions such as institution of declination statements, adding influenza vaccination to financial incentive systems, or vaccination as prerequisite for recruitment [12, 29, 31] should be considered to increase influenza vaccination coverage rates among HCWs.
